# Kidney-Gut Axis in Chronic Kidney Disease: Therapeutic Perspectives from Microbiota Modulation and Nutrition

**DOI:** 10.3390/nu17121961

**Published:** 2025-06-09

**Authors:** Shu Wakino, Kazuhiro Hasegawa, Masanori Tamaki, Masanori Minato, Taizo Inagaki

**Affiliations:** Department of Nephrology, Institute of Biomedical Sciences, Tokushima University Graduate School, Tokushima 770-8503, Japan

**Keywords:** chronic kidney disease, kidney-gut axis, microbiota, diet, intervention

## Abstract

Chronic kidney disease (CKD) has a high prevalence worldwide, with an increasing incidence. One of the mechanisms of CKD progression involves a disordered inter-organ relationship between the kidneys and the intestine, known as the kidney-gut axis. In CKD, two pathological gut conditions—disturbed gut microbiota composition called uremic dysbiosis and leaky gut—contribute to the progression of CKD. Dysbiosis is associated with the increased production of gut-derived uremic toxins, leaky gut, and chronic systemic inflammation, leading to worsening uremia, which in turn aggravates the gut condition. This vicious cycle should be a target of the therapeutic strategy against CKD. The modulation of uremic dysbiosis, including prebiotics, probiotics, and synbiotics, has been a typical treatment approach, although clinical evidence for their efficacy has been insufficient. Some non-antibiotic drugs have an impact on human gut bacteria that are believed to play a role in their clinical efficacy on kidney function. Nutrition therapies, including a low-protein diet, dietary fiber, a Mediterranean diet, and whole grains, positively influence gut microbiota composition and have been linked to a decreased risk of CKD. Novel strategies are currently being explored, involving the use of postbiotics, microbiome sequencing techniques, and fecal microbiota transplantation, although clinical application remains to be tested. Human trials investigating the above-mentioned interventions remain inconclusive due to several limitations, including dietary variability and genetic factors. Future research should focus on the development of more effective probiotics, prebiotics, and microbial metabolism-modifying drugs, not only for CKD but for other systemic diseases as well.

## 1. Introduction

**Figure 1:**
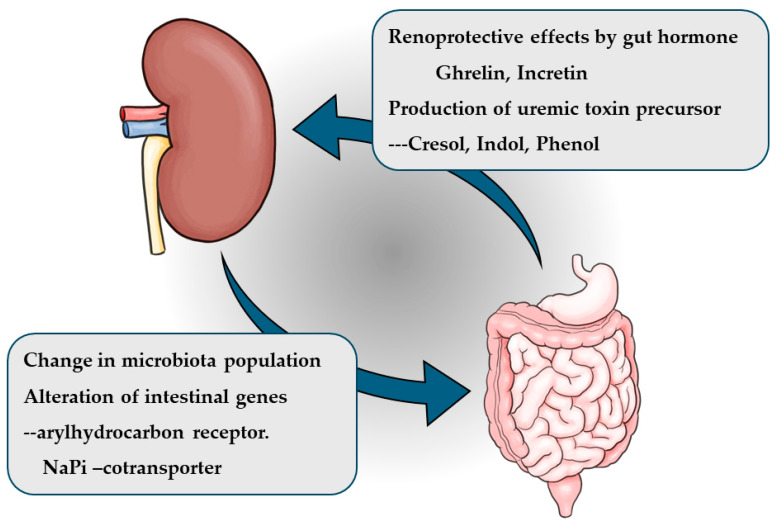
Kidney-gut axis.

In recent years, the inter-organ relationship between the kidneys and intestines has gained attention. This relationship, known as the kidney-gut axis, involves the action of intestinal hormones such as ghrelin and incretin on the kidneys, as well as the impact of uremic toxin absorption on kidney function. Additionally, studies have shown that the expression of certain intestinal genes and the composition of the intestinal microbiome are altered in chronic kidney disease (CKD). Similar changes in the intestinal microbiota have also been reported in patients with end-stage kidney disease. The kidney-gut axis represents a novel inter-organ relationship centered on the kidneys and is increasingly being recognized as a potential therapeutic target for CKD [1] (Figure 1). This review discusses the kidney-gut axis based on findings from previous studies.

## 2. Methods

This review was written as a narrative review, documenting the evidence for the inter-organ relationship between the kidneys and the gut in individuals with CKD. In part, a systematic literature search was performed on the descriptions mentioned in Section 5, titled ‘Nutritional therapy for kidney-gut axis’, mainly across PubMed to identify relevant studies published between 2013 and 2024. Studies were selected based on the inclusion criteria, which consisted of randomized controlled trials (RCTs), meta-analyses, systematic reviews, clinical guidelines, and expert recommendations regarding the impact of each nutritional therapy on kidney function and microbiota population.

## 3. Kidney-Gut Axis and Intestinal Microbiota

**Figure 2:**
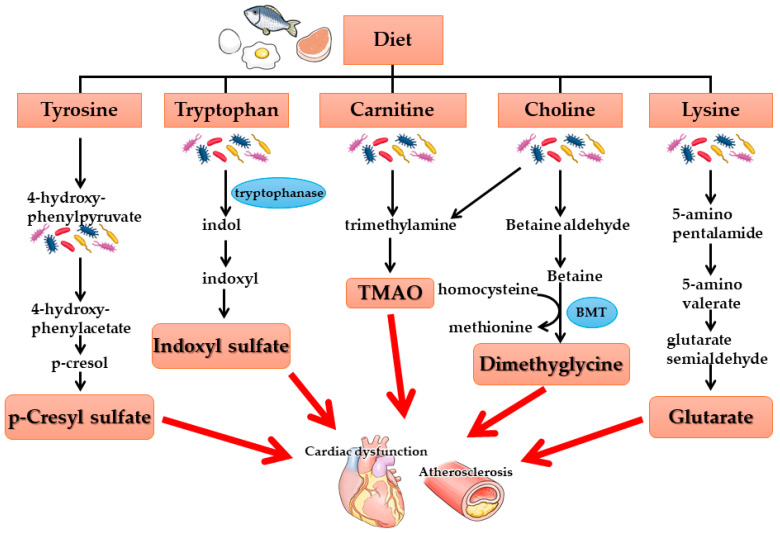
Gut-derived uremic toxins–gut microbiota metabolites. BMT: betaine methyltransferase.

As kidney function declines, uremic toxins produced by intestinal bacteria accumulate in the body. Several of these toxins have been identified, including *p*-cresyl sulfate (pCS), indoxyl sulfate (IS), trimethylamine-N-oxide (TMAO), dimethylglycine, and glutaric acid, all of which have been associated with cardiovascular events (Figure 2). Among these, IS and pCS are known to increase oxidative stress in cardiovascular tissues, leading to tissue damage. These uremic toxins are thought to originate in the intestinal tract due to alterations in the gut microbiota associated with kidney failure. Furthermore, intestinal permeability has been shown to increase in kidney failure, a process regulated by tight junction proteins such as claudin, occludin, and zonula occludens-1 (ZO-1), which maintain epithelial integrity. In CKD, the expression of these tight junction proteins is downregulated.

**Figure 3:**
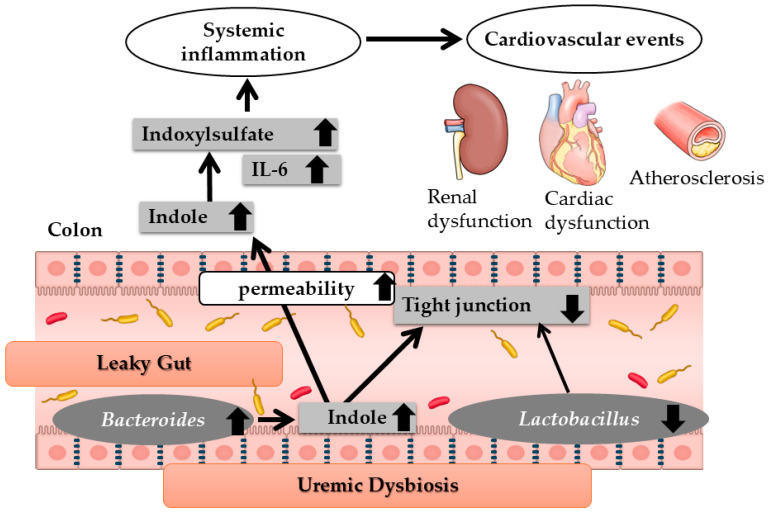
Changes in the intestinal environment in renal failure. IL-6; interleukin-6. Modified Ref. [2]. Thin arrows mean molecular changes or causal link. Thick arrows mean the increase or the decrease in molecules or microbiota.

In a rat model of chronic kidney failure, an increase in *Bacteroides* species was observed in the intestinal tract, along with an elevated production of indole, a precursor of the renal toxin IS. Conversely, the abundance of *Lactobacillus* species decreased, leading to reduced toll-like receptor expression and a subsequent decline in tight junction protein levels. This reduction in tight junction proteins resulted in increased intestinal permeability, facilitating the systemic absorption of indole, which is subsequently converted to IS in the liver and contributes to systemic inflammation. Elevated IS levels are associated with increased cytokine production, particularly interleukin-6 (IL-6), which is implicated in the heightened risk of cardiovascular complications in CKD [2] (Figure 3). Thus, two key pathological changes in the gut—uremic dysbiosis and increased intestinal permeability (leaky gut)—may contribute to cardiovascular events in kidney failure.

Several potential mechanisms for dysbiosis have been considered. One is the presence of higher concentrations of urea or uric acid in the blood in progressive renal failure. Exposure of intestinal bacteria to urea through intestinal secretions results in the conversion of urea to ammonia via bacterial urease. This high concentration of urea causes the overgrowth of bacterial families containing urease. Expansion of bacterial families producing uricase and indole- and *p*-cresyl-forming enzymes occurs in patients with end-stage renal disease (ESRD) compared with healthy controls [3]. Similarly, the increased secretion of uric acid in CKD accounts for the expansion of bacterial species that produce uricase [3]. However, in humans, these changes cannot isolate the effect of uremia from inter-individual variations, comorbid conditions, and dietary and medicinal interventions. Vaciri et al. used the experimental CKD rat model and showed that 5/6 nephrectomy induced a significant difference in the abundance of bacterial composition compared with control animals [4]. These sets of experimental evidence support the conclusion that dysbiosis is an effect of CKD.

The gut microbiota also plays a protective role against kidney damage through the production of metabolites such as short-chain fatty acids (SCFAs) [5]. SCFAs, including acetate, butyrate, and propionate, are generated through the saccharolytic fermentation of indigestible dietary fibers by gut bacterial enzymes. These SCFAs serve as an energy source for intestinal epithelial cells, help maintain the intestinal barrier, and promote the differentiation and proliferation of regulatory T cells (Tregs), which suppress inflammation and modulate intestinal immunity [6]. SCFAs also enhance the secretion of glucagon-like peptide-1 (GLP-1), a gut-derived hormone that regulates glucose and lipid metabolism and exerts anti-inflammatory effects [5,7]. Chronic systemic and renal inflammation play a key role in CKD progression, and the immunomodulatory properties of SCFAs may contribute to kidney protection. Studies using animal models of acute kidney injury have reported that SCFA administration mitigates kidney damage [8]. However, in CKD, the abundance of SCFA-producing gut bacteria declines due to dietary restrictions on potassium-rich foods and competition with toxin-producing microbiota, thereby exacerbating disease progression. Previous studies have reported that, in addition to an increase in uremic toxin-producing bacteria, the populations of SCFA-producing species such as *Lactobacillus*, *Prevotella*, and *Bifidobacterium* are reduced [4,9]. These changes have been attributed to various factors, including uremic toxin accumulation, metabolic acidosis, the effects of oral iron preparations and chelating agents on gut function, ischemia-induced intestinal dysfunction, and prolonged intestinal transit time due to inadequate fiber intake and constipation, both of which are commonly observed in CKD. Recent studies using a mouse CKD model have identified a novel pathway through which *Faecalibacterium prausnitzii* may contribute to kidney function restoration. This effect has been linked to butyrate-mediated activation of G-protein-coupled receptor 43 (GPR-43) signaling in the kidneys, highlighting a potential therapeutic avenue for CKD management [10].

## 4. Treatment Strategies Targeting Kidney-Gut Axis in CKD

### 4.1. Prebiotics, Probiotics, and Synbiotics

The treatment of CKD targeting the kidney-gut axis aims to ameliorate uremic dysbiosis and reduce uremic toxins produced by intestinal bacteria. This approach has been explored for a long time and includes prebiotics, which promote the growth of beneficial intestinal bacteria; probiotics, which directly replenish beneficial bacteria; and synbiotics, which combine both strategies. Although these treatments lower blood levels of uremic toxins such as indoxyl sulfate (IS) and *p*-cresyl sulfate (pCS), limited evidence supports their ability to improve kidney-related outcomes, including kidney function and survival [2,11]. Recent studies have investigated the effects of probiotic supplementation in patients with CKD stage G3 (CKDG3) aged 18 years and older. In an open-label, placebo-controlled trial, a 3-month intervention with *Lactobacillales* and *Bifidobacteria* improved iron kinetics, inflammatory parameters, and lipid metabolism but had no significant effect on estimated glomerular filtration rate (eGFR) [12]. Similarly, a 12-week probiotic supplementation using *Lactobacillus acidophilus*, *Lactobacillus casei*, and *Bifidobacterium bifidum* in patients with diabetes undergoing hemodialysis improved glucose homeostasis parameters and certain biomarkers of inflammation and oxidative stress. Although the treatment improved the Subjective Global Assessment (SGA) score, which is an indicator of subjective nutritional status, it did not significantly affect kidney function [13].

The supplementation of SCFA-producing microbiota and the modulation of renal dysbiosis appear to be promising therapeutic approaches for slowing CKD progression. Several recent clinical trials have been conducted, but while probiotics reduce uremic toxin levels, they do not appear to improve kidney outcomes. The ProLowCKD study, conducted in Italy in 2022, evaluated the addition of probiotics to a low-protein diet (0.6 g/kg body weight) in patients with CKD and an eGFR of <25 mL/min/1.73 m^2^ over 12 weeks. In the probiotic group, the number of urinary toxins decreased, and the doses of antihypertensive drugs and diuretics were reduced. However, no significant differences were observed between the groups in urinary protein excretion or eGFR. Although there was no difference in the SF-36 quality-of-life measure, the probiotic group showed an improvement in emotional role functioning [14]. In the SYNERGY II study, 68 adults with CKD and an eGFR of 15–60 mL/min/1.73 m^2^ were randomized to receive synbiotic therapy (*n* = 35) or placebo (*n* = 33) for 12 months. Synbiotic supplementation altered the stool microbiome by increasing *Bifidobacterium* and *Blautia* spp.; however, this was associated with a decline in eGFR. No significant differences were observed between the groups in cardiovascular structure and function, serum IS and pCS levels, blood pressure, or lipid profile [15].

Recent meta-analyses have provided limited evidence supporting the use of prebiotics, probiotics, or synbiotics in CKD management. A meta-analysis of 16 randomized controlled trials (RCTs) in CKD populations, with sample sizes ranging from 9 to 124 patients and intervention durations of 1–24 weeks, found little to no effect on serum urea, IS, or pCS levels. Synbiotic intervention significantly increased *Bifidobacterium* abundance, but its clinical implications remain unclear [16]. Another meta-analysis of RCTs in patients on dialysis, with sample sizes ranging from 15 to 98 patients and intervention durations of 4–24 weeks, reported that probiotic, prebiotic, and synbiotic supplementation significantly reduced serum C-reactive protein and interleukin-6 levels while increasing high-density lipoprotein cholesterol levels [17]. However, another recent meta-analysis demonstrated a favorable influence of biotics on circulating markers of creatinine, oxidant stress, inflammation, and uremic toxins in patients with CKD [18]. These contradictory analyses, despite some positive outcomes, still offer promise for the clinical application of biotic therapy and the clinical translation of the kidney-gut axis. The underlying mechanisms remain unclear, and study durations or populations may have been insufficient to demonstrate meaningful effects on kidney function. Additionally, the included studies varied widely in probiotic strains and dosages, contributing to inconsistent clinical outcomes. Consequently, probiotics are currently considered an adjunctive treatment strategy for kidney failure. Based on this disappointing evidence, several revised plans have already been raised. The length of the clinical trial should be longer. The selection of probiotic strains for human trials must be based on in vitro and in vivo scientific evidence. The selection of the best combination of synbiotics remains to be investigated. One missing piece we should consider is that the kidney-gut axis might be just one pivotal player in CKD pathophysiological conditions. For instance, the unfavorable effects of accumulated gut-derived uremic toxins result not only from their overproduction due to uremic dysbiosis but also from other factors, including decreased urinary excretion and intracellular accumulation through activation of the receptors for these toxins. Another concern is that uremic conditions other than the accumulation of gut-derived substances also play a role in the progression of CKD, including CKD-MBD disorder, renal anemia, and disturbed acid-base balance. Modulating the microbiota might have a limited effect, which would be necessary but insufficient to ameliorate CKD progression. More whole changes through nutrition therapy, which involve the amelioration of dysbiosis, are required.

### 4.2. Impact of Non-Antibiotic Drugs on Human Gut Bacteria

Several medications with prebiotic effects have been reported. Numerous non-antimicrobial drugs have also been shown to alter the intestinal microbiota [19]. In this study, we investigated the effects of the sodium–glucose co-transporter (SGLT) 2 inhibitor canagliflozin and the activated carbon adsorbent AST-120.

SGLT2 inhibitors are hypoglycemic drugs that have been in use for nearly a decade. These agents inhibit the activity of SGLT2, a sugar reabsorption transporter expressed in the kidneys, thereby increasing urinary glucose excretion and lowering blood glucose levels. Among SGLT2 inhibitors, canagliflozin has relatively low selectivity for SGLT2 and exhibits a weak inhibitory effect on SGLT1 compared with other agents in this class. As SGLT1 is primarily expressed in the small intestine, canagliflozin may also impair glucose absorption at this site. Therefore, we hypothesized that inhibiting glucose absorption in the small intestine would lead to fluctuations in the intestinal microbiota by increasing glucose delivery to the large intestine. To test this hypothesis, we examined changes in the gut microbiota following canagliflozin administration in nondiabetic 5/6 nephrectomy rats [20]. Following canagliflozin administration, a significant increase in colonic luminal glucose concentration was observed. Analysis of cecal stool using the terminal restriction fragment length polymorphism (T-RFLP) method confirmed that *Lactobacillus* levels decreased in rats with kidney failure but increased with canagliflozin treatment. PCR with bacteria-specific primers yielded similar results, showing a reduction in *Lactobacillus* spp. in rats with kidney failure and an increase following canagliflozin administration. Additionally, the expression of the intestinal tight junction proteins claudin-1 and occludin was reduced in nephrectomized rats but increased following canagliflozin treatment. In these rats, enhanced substrate availability promoted the proliferation of lactic acid bacteria involved in glucose fermentation, which is thought to ameliorate uremic dysbiosis and leaky gut. Furthermore, blood levels of indoxyl sulfate and hippuric acid, key uremic toxins, were reduced, and the extent of myocardial fibrosis, which was elevated in rats with kidney failure, was significantly reduced with canagliflozin treatment. Expression levels of fibrosis markers, connective tissue growth factor, and transforming growth factor beta were elevated in the hearts of rats with kidney failure but were reduced following canagliflozin administration. Histological staining results suggest that canagliflozin exerts an inhibitory effect on myocardial fibrosis. Additionally, in the kidney failure group, thoracic aortic vessel wall thickness was increased, whereas in the canagliflozin-treated group, the vascular wall thickening was mitigated. A reduction in blood indoxyl sulfate concentration following canagliflozin administration is thought to have contributed to the suppression of arteriosclerosis and led to the improvement in myocardial fibrosis.

Effects of the activated carbon adsorbent AST-120 on the intestinal environment were also investigated. Similar studies have been conducted in rats with kidney failure [21]. Analysis of the intestinal microbiota in cecal stool revealed an increase in Bacteroides in the kidney-insufficiency group; however, AST-120 administration did not induce significant changes in Bacteroides abundance. In contrast, Lactobacillus levels, which were significantly reduced in the kidney-insufficiency group, increased following AST-120 treatment. Furthermore, the expression of the tight junction proteins occludin, ZO-1, and claudin-1, which was significantly reduced in the kidney-insufficiency group, improved following AST-120 administration. Additionally, the proportion of goblet cells producing mucin in the intestinal crypts was lower in the kidney-insufficiency group but significantly increased with AST-120 treatment. These findings suggest that AST-120 modifies the intestinal environment, potentially by reducing intestinal indole levels or altering the mucin layer, thereby restoring Lactobacillus abundance and tight junction protein expression. As a result, blood concentrations of indoxyl sulfate and pro-inflammatory cytokines decreased, which might have positively influenced kidney function [21]. However, evidence supporting the effects of AST-120 on kidney function remains limited. A placebo-controlled randomized controlled trial of AST-120 in patients with CKD (EPPIC trial) found no significant effect on the mean change in eGFR from baseline [22]. Several potential reasons for these negative findings have been proposed, including discrepancies between actual and estimated placebo event rates, the need for a longer study duration, regional differences in dialysis initiation, and poor compliance with AST-120 treatment. Conversely, a recent randomized controlled trial of AST-120 in patients with deteriorating kidney function was conducted, enrolling 226 patients in the treatment group and 239 in the control group over a 3-year period. The results demonstrated that AST-120 slowed the rate of eGFR decline [23]. As an absorbent phosphate-binder, sevelamer, affecting the intestinal condition, was reported to reduce the serum concentration of a gut-derived uremic toxin, *p*-cresol, suggesting its beneficial effects on inflammation in patients with CKD [24]. In addition to our study, another investigation examined microbiota alterations associated with the SGLT2 inhibitor AST-120, suggesting that some of its beneficial effects in patients may be mediated through modifications in the gut microbiota [25,26].

Herein, two drugs, SGLT2 inhibitor and activated carbon adsorbent AST-120, were introduced as non-antibiotic drugs that affect the human microbiome. These examples span a broad range of drugs, not only for CKD-targeted drugs, and include antidiabetic metformin, nonsteroidal anti-inflammatory drugs, antipsychotics, proton-pump inhibitors, laxatives, and statins. The microbiota shifts induced by these drugs are not necessarily unfavorable, but rather enhance the drug efficacy in some cases. These kinds of investigations elucidating drug-microbiome-host interactions are just beginning to gain attention, and may, in the future, will optimize drug therapies, help develop new drugs, and shed light on the potential role of the microbiome in drug–drug interactions [27].

## 5. Nutritional Therapy for Kidney-Gut Axis

### 5.1. Low-Protein Diet

Dietary composition influences the gut microbiota, and the effects of protein-restricted diets have been investigated [28]. Several studies have reported an increase in *Lactobacillaceae*, *Bacteroidaceae*, and *Streptococcus anginosus*, which are short-chain fatty acid (SCFA)-producing bacteria expected to confer beneficial effects. Additionally, a decrease in *Bacteroides eggerthii* and *Roseburia faecis* has been observed, potentially leading to a reduction in uremic toxin levels. However, no evidence to date supports that a protein-restricted diet directly reduces uremic toxin levels, improves kidney function, or lowers C-reactive protein (CRP) levels by altering the gut microbiota. Furthermore, the clinical significance of these microbial changes remains unproven [29,30,31,32].

Since a protein-restricted diet itself has favorable effects on the microbiota population, the combination with interventions targeting intestinal bacteria has been tested. First, a study from India in 2016 reported results in which synbiotic tablets were administered to patients with stage 3–5 CKD following a protein-restricted diet (<0.6 g/kg standard body weight/day). The synbiotic tablets contained 15 × 10^9^ CFU of various bacteria (*Streptococcus thermophilus*, *L. acidophilus*, and *Bifidobacteria longum*) and 100 mg of fructooligosaccharides. No change was noted in serum creatinine levels [33]. In a study conducted in Italy involving patients with stage 3–4 CKD, a protein-restricted diet (0.6 g/kg standard body weight/day) with 19 g/day of inulin was administered for 6 months, and the quality-of-life assessment scale SF-36 (36-Item Short-Form Health Survey) showed improvements in daily life functions and overall health perceptions [34]. A similar study conducted at another facility in Italy showed metabolic effects and an improvement in the functional status indices on the SF-36 without any effects on kidney function [35]. A study from South Africa reported on the effects of β-glucan, where an intervention group taking a 13.5 g β-glucan prebiotic fiber supplement (GlucaChol-22^®^) daily with dietary protein restriction to 0.8 g/kg body weight/day showed a decrease in blood uremic toxins compared to the control group despite no significant differences in kidney function [36]. The aforementioned clinical trials, the ProLowCKD study [14] and the SYNERGY study [15], used a combination intervention consisting of a protein-restricted diet (0.6 g/kg standard weight/day) and synbiotic therapy, and failed to show any efficacy for kidney function.

There have been some concerns regarding this protocol that low-protein diets aggravate malnutrition or a protein–energy–wasting condition in individuals with CKD [37]. However, this assumption has been negated in clinical studies when applied to metabolically stable subjects with ketoanalog supplementation [38,39,40,41,42] and without ketoanalog supplementation [43,44,45], or even in an aged population [38,43]. The reason for this safety profile is that a low-protein diet ameliorates metabolic acidosis or insulin resistance [46], which protects against muscle degradation and frailty in patients with CKD. Moreover, if dietary protein restriction is practiced with adequate energy intake, sufficient energy does not lead to the degradation of amino acids for energy production via ketogenesis and glucogenesis pathways [47] and maintains muscle protein. Therefore, protein restriction should be prioritized in CKD individuals without poor nutritional status and personalized as part of a tailored nutritional intervention [48]. Once it is introduced, their nutrition status and energy intake should be monitored regularly.

Although the effects on kidney function are currently convincing [38,40,41,42,43,44,45], the potential negative impact on some frail CKD individuals cannot be ignored. However, a protein-restricted diet is worth applying in clinical practice. In this case, the protein-restricted diet is better prescribed as a combination therapy with some kind of synbiotic intervention since its metabolic and/or functional effects can be expected.

### 5.2. Fiber Diet

Dietary fiber is defined by the Food and Agriculture Organization/World Health Organization as nondigestible carbohydrates found in grains, seeds, vegetables, and fruits. The European Food Safety Authority defines dietary fiber as indigestible, nonabsorbable carbohydrate polymers and lignin that provide health benefits. Dietary fibers are classified into the following four types: (i) non-starch polysaccharides, (ii) indigestible polysaccharides, (iii) indigestible starch, and (iv) lignin. The characteristics of each group are summarized in Table 1 [49].

Dietary fiber is beneficial for overall health, with one key advantage being its role in bowel motility. Viscous fibers form a gel upon contact with water, softening stool and stimulating intestinal cells to promote peristalsis. More importantly, fiber serves as an energy source for saccharolytic bacteria, which ferment sugars and produce SCFAs. Intestinal bacteria can be categorized as saccharolytic bacteria, which metabolize carbohydrates, and proteolytic bacteria, which metabolize proteins and amino acids. Saccharolytic bacteria are considered beneficial, as they generate SCFAs that provide energy to intestinal cells, enhance gut barrier function, support immune responses, and exhibit systemic anti-inflammatory effects. Additionally, they suppress the growth of proteolytic bacteria, which produce putrefactive metabolites such as indoxyl sulfate, *p*-cresol, and TMAO. These metabolites promote inflammation and accumulate in patients with CKD. The suppression of proteolytic bacterial proliferation is a major benefit of dietary fiber. The anti-inflammatory properties of dietary fiber have been epidemiologically demonstrated. The NHANES III study reported that for every 10 g increase in dietary fiber intake, CRP levels decreased by 11% in healthy individuals and by 38% in patients with CKD. However, fiber intake in patients with CKD was found to be approximately 15.4 g/day, substantially lower than the US recommendation of 14 g/1000 kcal [47]. The recommended daily fiber intake is 20–35 g for healthy individuals, but intake remains insufficient among the Japanese population. Consequently, the Japanese Ministry of Health, Labour and Welfare has set daily fiber intake targets at 21 g for adult men and 18 g for adult women [49,50].

Epidemiological studies have revealed an inverse association between the intake of dietary fiber and CKD incidence [51]. Across three cohorts, individuals with the highest fiber intake exhibited a 40–50% lower incidence of CKD than those with the lowest intake. A meta-analysis of seven clinical studies on diabetic kidney disease reported that vegetarian diets were associated with a lower incidence of albuminuria and higher eGFR levels as compared with Western diets [52]. Prebiotics and synbiotics, such as inulin and resistant starch, have been investigated for CKD treatment. In CKD stages G3–G4, short-term lactulose supplementation was found to increase *Bifidobacteria* and *Lactobacillus* populations. Dietary fiber containing indigestible starch, arabino-xylo-oligosaccharide, and gum acacia (gum arabic) has demonstrated beneficial effects, including improved kidney function and reduced CKD complications. Meta-analyses have further shown that dietary fiber intake reduces serum creatinine levels and concentrations of uremic toxins such as *p*-chlorosulfate (pCS) and indoxyl sulfate (IS) [53,54,55]. However, no interventional studies have conclusively demonstrated a role in slowing CKD progression.

In dietary therapy for kidney failure, fruit and vegetable consumption is often limited due to potassium restriction, leading to low dietary fiber intake. However, recent dietary recommendations allow for controlled fruit and vegetable intake, as studies have reported weak or no correlation between serum potassium levels and dietary potassium intake in end-stage kidney disease, including patients on dialysis. Moreover, patients on dialysis who consume more vegetables and fruits tend to have lower mortality rates [56]. Potassium absorption and serum potassium levels are influenced by multiple factors. For instance, when ingested with glucose or fructose, potassium is rapidly transported into cells [57]. Dietary fiber intake has also been associated with increased fecal potassium excretion due to enhanced stool bulk in patients with CKD [58]. Given the presence of fructose, fiber, and alkalizing agents in fruits and vegetables, their consumption is unlikely to elevate serum potassium levels. Hence, generalized restrictions on fiber, vegetable, and fruit intake may be counterproductive [59].

Among dietary fibers, resistant starch (RS) has garnered attention. RS is a category of starch that resists digestion in the small intestine and reaches the colon, where it undergoes fermentation. RS is classified into five subtypes (RS1–RS5), as summarized in Table 2. Experimental CKD models have demonstrated that RS promotes the growth of *Bifidobacteria* and *Lactobacilli*, enhances SCFA production, and reduces intestinal uremic toxin generation [60]. In clinical studies on patients with CKD, RS supplementation altered gut microbiota composition in patients on hemodialysis but did not lower systemic uremic toxin concentrations [60].

Consistent with these findings, several human studies have investigated RS supplementation in patients with CKD. Some studies have reported biochemical improvements in patients on dialysis [61,62,63,64,65,66,67,68,69]. A meta-analysis concluded that RS supplementation reduces uremic toxin levels and improves kidney function but does not attenuate inflammation in patients with CKD [70].

Despite its benefits, dietary fiber intake may have drawbacks, including deficiencies in amino acids, vitamin B12, iron, and zinc [71]. Additionally, unhealthy vegetarian diets often contain highly processed foods, preservatives, and additives, which may be detrimental to patients with CKD [72]. Consequently, specialized dietary interventions should be personalized in consultation with a registered dietitian.

### 5.3. Whole Grains

Whole grains refer to grain kernels rich in dietary fiber, phytochemicals, minerals, antioxidants, vitamins, and other beneficial nutrients [73]. A high intake of whole grains has been widely recognized for its potential to reduce the risk of type 2 diabetes, coronary heart disease, and cancer [74,75,76]. Compared with refined grains, whole grains positively influence gut microbiota composition and exert anti-inflammatory and antioxidant effects, contributing to the prevention of various diseases [77,78]. A lower intake of whole grains has been linked to an increased risk of CKD [73,79]. Recent population studies have demonstrated that a higher whole grain intake relative to refined grains is associated with a lower risk of cardiovascular disease, kidney failure, and mortality in patients with CKD. Notably, patients with CKD are prone to constipation, partly due to gut microbiota dysbiosis, which may be alleviated by fiber-rich whole grains. A large cohort study reported an association between constipation and CKD prevalence and progression [80]. Whole grains have the potential to prevent or slow CKD progression.

Rice is a staple food, particularly in Asia, and one of the most consumed grains worldwide. During rice production, rough rice undergoes threshing to remove the husk, yielding brown rice, which can be further processed through milling to remove the bran and produce white rice [81]. Brown rice is a whole grain rich in bioactive compounds such as flavonoids, phenolics, vitamins, phytosterols, and oils. These compounds are concentrated in the bran layer of brown rice. Given the favorable effects of whole grains on CKD and the anti-inflammatory and antioxidant properties of brown rice [82], its consumption is expected to mitigate CKD progression.

With limited evidence from clinical trials, the effects of whole grains, including brown rice, on kidney function remain to be investigated. Moreover, with regard to brown rice, the bran layer, which is to be removed in usual white rice, is rich in protein, phosphorus, and potassium and can be hazardous to patients with CKD. To overcome these disadvantages, low-protein brown rice has been developed and is being tested in a multicenter clinical trial in Japan [83].

### 5.4. Mediterranean Diet and Other Healthy Dietary Patterns

The Mediterranean diet (MD) consists of vegetables, fruits, fish, olive oil, nuts, germinating grains, and moderate amounts of red wine. It has a high content of fermentable carbohydrates, vegetable proteins, and fish oil. This dietary pattern is rich in unsaturated fatty acids and is associated with several health benefits. In contrast, the Western diet (WD) includes red meat, processed foods, refined grains, and high amounts of sugar, salt, phosphorus, animal protein, and saturated fat. Studies have shown that the intestinal microbiota composition differs between MD and WD [4]. The MD promotes the proliferation of saccharolytic bacteria, whereas the WD favors the growth of proteolytic bacteria, leading to microbial imbalance. Moreover, a recent observational, cross-sectional study revealed that the MD pattern as assessed by aMED score was associated with lower gut-derived uremic toxin, pCS levels in patients on dialysis, which suggested the MD can mitigate the uremic condition of chronic kidney disease and that MD can be a potential therapeutic strategy against CKD comorbidities by modulating the kidney-gut axis [84]. Several studies have reported that MD suppresses the onset and progression of CKD; however, the available evidence remains inconclusive [85].

Clinical evidence supporting the efficacy of MD is limited, with few randomized controlled trials reported thus far. In a recent study, 1002 patients with coronary heart disease and an estimated eGFR of ≥30 mL/min/1.73 m^2^ were randomized to follow either an MD or a low-fat diet. The MD resulted in a lower eGFR decline among patients with obesity and type 2 diabetes compared with the low-fat diet. Additionally, the MD reduced urinary albumin excretion only in this population [86]. Another randomized crossover trial has demonstrated that the Mediterranean Proper Optimal Balance (MEDi-POB) diet did not adversely affect serum and urine potassium levels and helped maintain kidney function in Korean patients with stage 3–4 CKD [87]. Recently, a modified Mediterranean dietary pattern adjusted for CKD individuals was proposed [88], named the MedRen diet. MedRen diet is distinguished from the MD in terms of the quantities of proteins, phosphorus, and salt, which are designed for CKD individuals. Although the clinical study and the impacts on the gut environment or dysbiosis have not been reported thus far, additive favorable effects of the low protein diet and MD would be expected.

Apart from the MD and MedRen diets, other dietary protocols have been established as healthy dietary patterns for CKD individuals. The PLADO diet (plant-dominant low-protein diet) is characterized by a protein intake of 0.6–0.8 g/kg/day, of which at least 50% is derived from plant-based sources. This diet is also characterized by avoiding ultra-processed foods, an adequate dietary energy intake (i.e., 30–35 Kcal/kg/day), and a low sodium intake. The flexitarian diet is also plant-based, but may occasionally contain small portions of fish, meat, and dairy products [89,90]. Currently, there have been no randomized controlled trials with PLADO or flexitarian dietary nutritional therapy on CKD individuals in the literature. Their effects on the gut microbiota or gut environment remain to be explored, although the increased consumption of foods rich in fiber, such as plant-based foods or whole grains, in patients with CKD by these newly proposed plant-based dietary patterns could ameliorate the balance of the gut microbiota [91,92].

There have been concerns about hyperkalemia, protein–energy wasting, and conflicting RCT results in a plant-based diet or MD. However, the risk of hyperkalemia is considered to be low because the intake of potassium from plant foods is associated with an alkalizing effect, the intake of fiber reduces the risk of constipation, and carbohydrate intake stimulates the release of insulin and the consequent entry of potassium into the cells, reducing the risk of developing hyperkalemia [93,94]. With regard to protein–energy wasting, among non-CKD [95] and CKD populations [96], higher consumption of fruit and/or vegetables was correlated with a reduced risk of sarcopenia. Finally, despite the lack of RCTs adopting a plant-based diet, recent observational studies support the favorable effects of a plant-based diet on CKD progression. One study, which enrolled nearly 15,000 people in the Atherosclerosis Risk in Community Study group, showed that high adherence to a plant-based diet was associated with a decreased incidence of CKD, as well as a decreased decline in kidney function over time [97]. In the Singapore Chinese Health Study, it was shown that substituting one serving of red meat with one serving of soy/legumes correlated with a lower risk of incident ESKD [98].

Although all the interventions to the kidney-gut axis through biotics, dietary fiber, and whole grains were not sufficiently successful, the change in dietary pattern, for instance, into a protein-restricted diet or MD, is efficacious for kidney function. The reason for this favorable effect is its multifaceted advantages for preventing CKD initiation and progression. MD harbors sufficient dietary fiber, whole grains, and growth-promoting effects on SCFA-producing saccharolytic bacteria. Its effects are sustainable when this dietary pattern continues in everyday meal intake. The only barrier against the MD intervention could be that the MD has been applied especially to Western countries. The corresponding dietary patterns of MD for Asian countries or Japanese subjects should be developed. The traditional Japanese style diet is supposed to be plant-based and uses fish as one of the main protein sources, resembling an MD pattern [99]. Therefore, in clinical practice, the shift or adherence to a traditional Japanese-style diet can be a plausible therapeutic strategy for Japanese patients with CKD.

### 5.5. Novel Strategies Targeting Kidney-Gut Axis

Postbiotics are emerging interventions composed of inactivated microorganisms and their fermented products. In 2019, the International Scientific Association of Probiotics and Prebiotics defined postbiotics as a “preparation of inanimate microorganisms and/or their components that confers a health benefit on the host” [100]. Preclinical studies have evaluated the effects of sonicated Lactobacillus paracasei in high-fat diet-induced kidney injury [101] and GABA-salt, lacto-GABA-salt, and postbiotic-GABA-salt in acute kidney injury [102]. Although the clinical application of this strategy in the CKD and dialysis field is still in the preclinical stage, one single-center non-randomized pilot study was reported where 20 maintenance dialysis patients were administered with SCFA, sodium propionate. The study showed that sodium propionate supplementation reduced pro-inflammatory parameters and oxidative stress and improved insulin resistance and iron metabolism [103].

The efficacy of gut microbiota-targeted therapies depends on inter-individual variability in diet, lifestyle, age, and medication use. To address these limitations, microbiome sequencing techniques are essential for enabling more precise and personalized interventions. A study analyzing gut microbial profiles using 16S rRNA sequencing compared healthy controls with patients with CKD, membranous nephropathy, immunoglobulin (Ig) A nephropathy, minimal change disease, and ischemic kidney injury. This study found a strong correlation between gut microbiome dysregulation and CKD subtypes [104]. Assessing the microbiome of each patient is crucial for selecting and monitoring dietary and biotic strategies tailored to CKD management [105].

Fecal microbiota transplantation (FMT) involves the transfer of gut microbiota from a healthy donor to a patient, aiming to restore the microbial balance. A study found that FMT reduced albuminuria and modulated kidney function in a mouse model of IgA nephropathy [106]. Recently, an RCT evaluated the effects of encapsulated fecal microbiota derived from healthy donors. Patients in the placebo group exhibited a higher rate of CKD progression than those in the FMT group. Moreover, the FMT group maintained stable kidney function, as indicated by serum creatinine and urea nitrogen levels, compared with the placebo group [107]. Although the clinical application of FMT to CKD or dialysis patients is still in the preclinical stage, one single-center, double-blind, randomized, placebo-controlled clinical trial was reported. Patients with CKD clinical stages 2–4 were randomly assigned to receive either FMT or placebo capsules for 6 months. After FMT administration, CKD patients showed less disease progression [107].

As already stated, these treatment strategies are still in their early stages and have not yet led to products or protocols for clinical use. Their feasibility or adaptability to clinical practice remains to be investigated.

## 6. Conclusions

This review explored the kidney-gut axis, therapeutic strategies, and clinical implications in CKD. A key finding is that *Lactobacillus* levels decrease in kidney failure, disrupting the intestinal barrier and increasing the invasion of uremic toxin precursors, such as indole, which contribute to kidney damage, cardiovascular disorders, and systemic inflammation. Several drugs influence gut microbiota composition and uremic toxin levels. Canagliflozin, an SGLT2 inhibitor, modulates intestinal microbiota and reduces uremic toxins, while AST-120 improves gut dysbiosis and promotes *Lactobacillus* recovery, thereby lowering the circulating toxin levels and potentially preserving kidney function. However, human trials investigating microbiota modulation remain inconclusive due to several limitations, including dietary variability (fiber and protein intake), antibiotics and medication use, population-specific microbiota differences, and genetic factors. Moreover, probiotic studies have used diverse bacterial strains and dosages, leading to uncertainties regarding the optimal formulation and dosage for CKD management. Dietary therapy, particularly in the context of the kidney-gut axis, is critical for CKD management. While food serves as a fundamental source of nutrition, impaired excretion and metabolism in CKD can transform certain dietary components into harmful agents. Nutritional therapy must account for intestinal dysfunction in CKD, as kidney failure alters the gut microbiota, absorption, and motility. This change should be considered in dietary therapy, and it can be stressed that nutritional therapy for kidney failure has entered a new stage. Future research should focus on the development of more effective probiotics, prebiotics, and microbial metabolism-modifying drugs, not only for CKD but for other systemic diseases as well.

Finally, we present the following takeaways for CKD clinical practice based on this review.

1. A disturbed kidney-gut axis should be considered in CKD clinical practice.
2. The partial correction of a disturbed kidney-gut axis by a single treatment bundle, including biotics therapy, protein-restricted diet, dietary fiber, and whole grains, is not effective in halting the progression of CKD, although it may have some favorable effects on patients’ quality of life.
3. The switch or shift to the healthy whole dietary pattern—involving the shift or adherence to a plant-based diet such as the MD and harboring multiple therapeutic strategies targeting the kidney-gut axis—is the most plausible measure for CKD treatment.

## Figures and Tables

**Table 1:** Classification of dietary fibers based on their physicochemical characteristics.

Classification	Fibers
Non-starch polysaccharide	Cellulose Hemicellulose Mannan Heteromannan Pectin Inulin Fractan
Resistant oligosaccharide	α-galactosides β-fructooligosaccharides (FOS) α-galactooligosaccharides (GOS) β-galactooligosaccharides (TOS) Xylo-oligosaccharides (XOS) Arabinoxylooligosaccharides (AXOS) Polydextrose
Resistant starch	Type 1—starch resistant to amylase Type 2—ungelatinized starch, powdered starch Type 3—recrystallized starch Type 4—chemically modified starch Type 5—complex of amyloses and lipids
Other dietary fibers	Lignin Chitins

**Table 2:** Classification of resistant starch.

RS Type	Description	Examples
RS1	Physically inaccessible	Coarsely milled grains or seeds, legumes
RS2	Ungelatinized starch	Raw potato, unripe banana, high-amylose maize starch
RS3	Retrograded starch	Cooked, cooled foods (potatoes, pasta, rice), corn flakes
RS4	Chemically modified starch	Cross-linked starch and octenyl succinate starch
RS5	Amylose–lipid complex	Stearic acid–complexed high-amylose starch

Citation from ref. [43].
